# JBMR Plus Reviewer List 2022

**DOI:** 10.1002/jbm4.10702

**Published:** 2022-12-14

The ASBMR and the journal Editors are incredibly thankful for the reviewers who volunteer their time for the vital mentoring role of peer reviewer. The following individuals have reviewed for JBMR Plus in the past year and have provided invaluable feedback to help improve submitted papers.

Data through November 13, 2021 – October 31, 2022

*Indicates 3 or more reviews completed

**Indicates 5 or more reviews completed

Cheryl L. Ackert‐Bicknell

Imranul Alam

Rajeev Aurora

Yangjin Bae

Kristen Beavers

Loren Berman

Neil Binkley

Robert Blank

Stephan Blüml

Roger Bouillon

Alan Boyde

Aline Bozec*

Robert Brommage*

Angela Bruzzaniti

Thomas Carpenter

Peggy Cawthon

Jia Chang

Naibedya Chattopadhyay

Jin‐Ran Chen

Blaine Christiansen

Cristiana Cipriani

Bart Clarke

Adi Cohen

Kelsey Collins

Bernard Cortet

Nicola Crabtree

James Cray

Sarah Dallas

Luis Del Rio

Jesus Delgado‐Calle

Paola Divieti Pajevic

Matthew Drake

Sophie Dream

Kate Duchowny

Gustavo Duque

William Edwards

Yasser El Miedany

Florent Elefteriou**

Roman Eliseev

Klaus Engelke

Kristine Ensrud

Erik Eriksen*

Judith Everts

Alberto Falchetti

Carlos R. Ferreira

Benjamin Frisch

Masafumi Fukagawa

Seiji Fukumoto

Rachel Gafni

Thomas Gardella

Damian Genetos

Francesca Gori

Giorgia Grassi

Matthew Greenblatt

Daniel Grinberg

Scott A. Guelcher

Anyonya Guntur

Long Guo

Sarah Hardcastle**

Nan Hatch

Frans Heyer

Hironori Hojo

Michael Holick

Kristin Holvik

Namki Hong

Keith A. Hruska

Steven Ing*

Carlos Isales

Masaru Ishii

Urszula Iwaniec

Suzanne Jan de Beur

Fjola Johannesdottir*

Rachelle Johnson

Melissa Kacena

Ivo Kalajzic

Johan Kälvesten

Galateia Kazakia

Ann Kearns

Christine Kern

Frank Ko

Shoichiro Kokabu

Rajiv Kumar

Nancy Lane

Bente Langdahl*

Lisa Langsetmo

Brian Lentle

Joohyun Lim

Eva Liu*

X. Sherry Liu

Joseph Lorenzo

Heather Macdonald

Sujay Madduri

Ansuyah Magan

Outi Makitie

Jennifer Manilay

Kim Mansky**

Claudio Marcocci**

Kevin Martin

Takamitsu Maruyama

Yuki Matsumura

Brya Matthews

Meghan McGee‐Lawrence**

Daniela Merlotti**

Megan Michalski

Petar Milovanovic

Salvatore Minisola

Sharon Moe

Meghan Moran

Melissa Morimoto

Christian Muschitz**

Ken‐ichi Nakahama

Barbara Obermayer‐Pietsch

William Obremskey

Noriaki Ono

Wanida Ono

Satoru Otsuru

Susan Ott

Nejmeddine Ouerghi

Andrea Palermo

Youn‐Soo Park

Shaopeng Pei

Lilian Plotkin

Marco Ponzetti

Ann Prentice

Sylvain Provot

David Reed

Mirko Rehberg

Ian Reid

Ann Rosenthal

Ryan Ross

Danielle Rux

Hajime Sasaki

Ernestina Schipani

Felix Schmidt

Nicole Sekel

Deborah E. Sellmeyer

Jad Sfeir*

Nick Shaw

M. Kyla Shea

Enisa Shevroja

Xing‐ming Shi

Natalie A. Sims

Cristina Sobacchi

Martha Somerman

René St‐Arnaud

Rick Sumner

Atsushi Suzuki

Pawel Szulc

Hanna Taipaleenmäki

Yoshifumi Takahata

Jianning Tao

Elena Tsourdi**

Jan Tuckermann

Mandy Turner

Robert Tycko

Yasuyoshi Ueki

Bram van der Eerden

Wim Van Hul

Anja Verhulst

Stuart Warden

Connie Weaver

Thomas Weber

Natalie Wee

Marc Wein

Robert Wermers

Jeremy Wilkinson

Rebecca Willcocks

Andrew Williams

Bart Williams

Joy Wu

Sumit Yadav

Tao Yang

Maria Zanchetta

Babette Zemel

Cristiano Zerbini

Hongyuan Zhang

Haibo Zhao

